# STINGRAY: system for integrated genomic resources and analysis

**DOI:** 10.1186/1756-0500-7-132

**Published:** 2014-03-07

**Authors:** Glauber Wagner, Rodrigo Jardim, Diogo A Tschoeke, Daniel R Loureiro, Kary ACS Ocaña, Antonio CB Ribeiro, Vanessa E Emmel, Christian M Probst, André N Pitaluga, Edmundo C Grisard, Maria C Cavalcanti, Maria LM Campos, Marta Mattoso, Alberto MR Dávila

**Affiliations:** 1 Laboratório de Biologia Computacional e Sistemas, Instituto Oswaldo Cruz (IOC), Fundação Oswaldo Cruz (FIOCRUZ), Avenida Brasil 4365, 21040-360 Rio de Janeiro, Rio de Janeiro, Brazil; 2 Laboratório de Protozoologia, Departamento de Microbiologia, Imunologia e Parasitologia (MIP), Centro de Ciências Biológicas (CCB), Universidade Federal de Santa Catarina (UFSC), Campus Universitário, Setor F, Bloco A, Trindade, 88040-970, Caixa Postal 476, Florianópolis, Santa Catarina, Brazil; 3 Laboratório de Doenças Infecciosas e Parasitárias (LDIP), Área de Ciências Biológicas e da Saúde (ACBS), Universidade do Oeste de Santa Catarina (Unoesc), Rua Getúlio Vargas 2125, Flor da Serra, 89600-000 Joaçaba, Santa Catarina, Brazil; 4 Pólo de Biologia Computacional e Sistemas, Instituto Oswaldo Cruz (IOC), Fundação Oswaldo Cruz (FIOCRUZ), Avenida Brasil 4365, 21040-360 Rio de Janeiro, Rio de Janeiro, Brazil; 5 Laboratório de Biologia Molecular de Parasitas e Vetores, Instituto Oswaldo Cruz (IOC), Fundação Oswaldo Cruz (FIOCRUZ), Avenida Brasil 4365, 21040-360 Rio de Janeiro, Rio de Janeiro, Brazil; 6 Laboratório de Genética Molecular de Microrganismos, Instituto Oswaldo Cruz (IOC), Fundação Oswaldo Cruz (FIOCRUZ), Avenida Brasil 4365, 21040-360 Rio de Janeiro, Rio de Janeiro, Brazil; 7 Laboratório de Bioinformática, Instituto Carlos Chagas (ICC), Fundação Oswaldo Cruz (FIOCRUZ), Avenida Algacyr Munhoz Mader, 3775, Cidade Industrial, 81350-010 Curitiba, Paraná, Brazil; 8 Instituto Militar de Engenharia (IME), Seção de Engenharia de Computação (SE-8), Praça General Tibúrcio 80, Praia Vermelha, Urca, 22290-270 Rio de Janeiro, Rio de Janeiro, Brazil; 9 Instituto de Matemática, Departamento de Ciência da Computação, Universidade Federal do Rio de Janeiro, Bloco C, CCMN, Sala E-2206, Ilha do Fundão, 21945-970 Rio de Janeiro, Rio de Janeiro, Brazil; 10 Instituto Alberto Luiz Coimbra de Pós-graduação e Pesquisa de Engenharia (COPPE), Universidade Federal do Rio de Janeiro, P.O. Box 68511, Ilha do Fundão, 21941-972 Rio de Janeiro, Rio de Janeiro, Brazil

**Keywords:** Genome, Annotation, Workflow, Next generation sequencing, Sanger, Data integration

## Abstract

**Background:**

The STINGRAY system has been conceived to ease the tasks of integrating, analyzing, annotating and presenting genomic and expression data from Sanger and Next Generation Sequencing (NGS) platforms.

**Findings:**

STINGRAY includes: (a) a complete and integrated workflow (more than 20 bioinformatics tools) ranging from functional annotation to phylogeny; (b) a MySQL database schema, suitable for data integration and user access control; and (c) a user-friendly graphical web-based interface that makes the system intuitive, facilitating the tasks of data analysis and annotation.

**Conclusion:**

STINGRAY showed to be an easy to use and complete system for analyzing sequencing data. While both Sanger and NGS platforms are supported, the system could be faster using Sanger data, since the large NGS datasets could potentially slow down the MySQL database usage. STINGRAY is available at http://stingray.biowebdb.org and the open source code at http://sourceforge.net/projects/stingray-biowebdb/.

## Findings

With the expansion of genomic, transcriptomic and proteomic data, the availability for both intra and inter-specific analyses of nucleotide and protein sequences has raised new levels of difficulty for scientists to understand, integrate and compare this ever increasing information. An important and long lasting problem is how to process and deal with large complex sequence files with distinct formats and using different tools that do not easily exchange data with each other. Thus, researchers must deal with dozens of sequence formats and a variety software packages to analyze nucleotide or protein sequences.

In order to ease such tasks, researchers have been using alternative strategies such as the development of custom *ad-hoc* scripts, sometimes even ignoring pre-existing generic modules (e.g. Bioperl, Biopython, Bioruby, Biojava). It has been widely used and has proved its efficacy for simple environments, however *ad-hoc* scripting often results in redundant work and code, difficulties to adapt, which reduces efficiency and is a more error-prone development. Furthermore, the intermediate files generated throughout the process are usually not properly stored and organized, generating a large number of files and versions that can potentially lead to errors in data processing, analyses and/or inferences.

Alongside, the use of database management systems have facilitated several tasks by enforcing integrity constraints, supporting transaction management, concurrent access control, structuring and integrating data into a single schema, and providing structured query languages (SQL), among others.

Another common problem faced by many researchers is the difficulty to handle the installation and working with Unix/Linux-based software, as well as the integration of them. Therefore, the development of user-friendly applications is becoming more common providing a uniform user interface to integrate all these programs with their inputs/outputs in scientific workflows making the annotation and functional analysis process painless to users.

There are several sequence and expression analysis workflows described, such as the EST (Expressed Sequence Tags) pipeline system [1], SABIA [2], GARSA [3], GATO [4], JUICE [5], and others for Next Generation Sequencing (NGS) data analysis, such as NGSPE [6], WEP [7] and DDBJ Pipeline [8]. However, none of these systems were designed to deal at once with EST or GSS (Genome Survey Sequences) data or from different sequencing platform as NGS or Sanger technologies in the same system. Furthermore, those available systems usually don’t include protein, phylogenetic and ontology-based analyze such as STINGRAY does. Available workflows usually require some adaptation to optimize performance for each user. For this reason we have designed a flexible workflow in which researchers can use or combine its different sequenced data (subsets of functionalities), according to their needs, in order to ease and turn less time consuming the annotation process, regardless of the size of the genomic dataset.

### STINGRAY purpose, development and management

**Figure 1:**
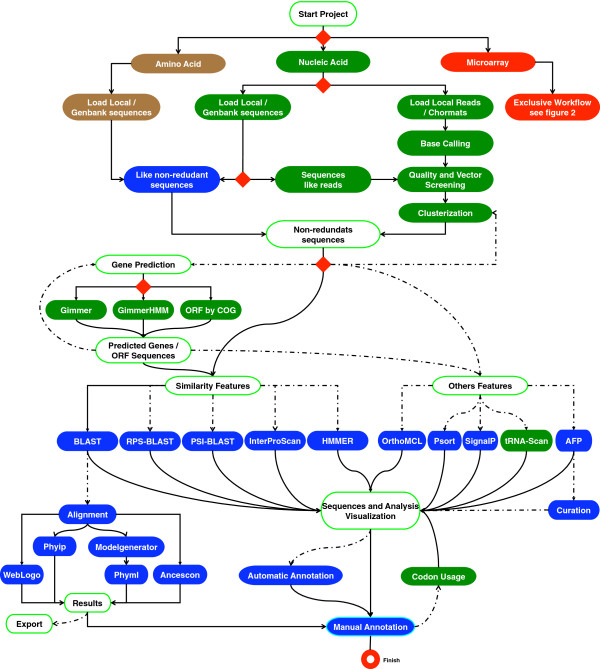
**Schematic representation of the STINGRAY workflow.** The white boxes show the analyses categories and respective visualization interface. Green boxes indicate software or features for nucleic acid analyses, brown boxes for protein analyses and blue boxes are for both. Red diamonds indicate the user decision or inputs points. Full lines represent minimal analyses and discontinuous lines are the alternative or non-obligatory analyses. The AFP box is the automatic functional prediction based on Gene Ontology.

Considering the previously mentioned challenges, plus the increase of available sequences and multi-team-based projects involving laboratories that are usually geographically dispersed, STINGRAY was conceived as an environment aiming to facilitate the storage, analysis, integration and presentation of genomic and gene expression information. This system integrates several bioinformatics tools and sequence databases, offering a flexible and user-friendly interface.STINGRAY workflow (Figure 1) was built upon the previous and smaller scale GARSA workflow and sustained significantly improvement as: (a) a larger number of bioinformatics programs; (b) automatic functional prediction and annotation; (c) improvement of phylogenetic analysis; (d) larger and more flexible workflow; (e) the use of a more comprehensive database schema; (f) connection with remote servers for intensive computing; (g) NGS datasets analysis; and (h) a user-friendly configuration interface, resulting in a new and comprehensive system.

The underlying STINGRAY platform includes Perl, Bioperl, CGI, Apache, MySQL, and several Linux-based bioinformatics packages (Table 1). In its current version, the system is able to handle EST, ORESTES and GSS Sanger, as well as NGS (454, SOLiD and Illumina) data, accepting as inputs: (a) Sanger-based chromatograms; (b) NGS-based 454′s flowgrams, Illumina’s FASTQ and SOLiD’s color space; (c) nucleotide or protein FASTA sequences from GenBank [9] (Additional file 1); (d) nucleotide or protein FASTA sequences stored locally; or (e) a combination of all of these inputs. Also, STINGRAY is able to analyze protein sequences, accepting both locally stored or sequences downloaded from GenBank, and to perform comprehensive sequence and genome analysis, distant homology detection and phylogenetic analysis.

**Table 1:** Bioinformatics software or packages incorporated on STINGRAY workflow

**Software/packages**	**Workflow function**	**Ref.**
MIRA	Assembly	[10]
Velvet	Assembly	[11]
Phred	Reads quality match	[12]
Crossmatch	Vector mask	[12]
RepeatMasker	Repeat sequence mask	[13]
CAP3	Sequences clusterization	[14]
Glimmer3	Prokaryotic gene prediction	[15]
GlimmerHMM	Eukaryotic gene prediction	[16]
Geecee	G + C content calculation	[17]
Cusp	Codon usage calculation	[17]
tRNA-scan	tRNA search	[18]
BLAST	Similarity search	[19]
Rps-BLAST	Conserved domain search	[19]
Psi-BLAST	Similarity search	[19]
Signalp	Signal peptide cleavage sites prediction	[20]
Wolf-Psort	Protein localization	[21]
MAFFT	Multiple sequence alignment construction	[22]
ProbCons	Multiple sequence alignment construction	[23]
WebLogo	Alignment logos generation	[24]
Ancescon	Ancestor sequence prediction	[25]
Phylip	Phylogenetic tree construction	[26]
Weighbor	Phylogenetic tree construction	[27]
ModelGenerator	Evolutionary model search	[28]

The STINGRAY system is being offered as a web server (i.e. CGI-based), so that common users do not need to deal with a large number of dependencies. A web-based setup page is available to configure dependency paths and other features (Additional file 1), thus eliminating the need for interacting directly with the Linux/Unix server. All programs (Table 1) can be configured to run locally (e.g. in the same server where STINGRAY is installed) or remotely, in a different server, like the structure available at Fundação Oswaldo Cruz - FIOCRUZ (Additional file 2).

Nowadays many researchers collaborate in the annotation process in different locations leading to control the different access grants for each user in order to avoid data loss, simultaneous modification, conflicts and security issues. STINGRAY system has data access control for six different user profiles: (a) system administrator; (b) project administrator; (c) "write" users (which can run programs and annotate sequences); (d) "read" users (which are not allowed to run programs or to do annotation); (e) "guest" users (which can only view non-sensitive data and low level of annotation details, and do not have permission to download/upload sequences); and (f) "statistics" users (which can only access statistical data about a project, e.g., total number of sequences analyzed).

### STINGRAY workflow

To provide an integrated view and execution of required tools, the current STINGRAY workflow has two major sections, (I) nucleotide and (II) protein. Both of them share the same initial configuration section (Figure 1). For nucleotide section, STINGRAY workflow uses the Phred [12] package to process chromatograms from Sanger technology, evaluating the traces quality and removing any occasional vector contamination. Following, Repeat Masker [13] is used to find and mask repeated sequences, and CAP3 [14] for clustering the sequences (reads) into a consensus sequence (clusters).

To deal with NGS datasets, MIRA [10] package is used to process 454 flowgrams [29] and Illumina reads, while ABI SOLiD™ System *de novo* Accessory Tools 2.0 package and VELVET [11] deal with the color-space dataset. These packages enable STINGRAY to perform the *de novo* assembly routines, provided by the short-read assemblers generating contigs data set and the output data can be loaded in STINGRAY databases that will consider the each sequence as cluster.

Gene predictions for prokaryote and eukaryote genomes are performed using Glimmer [15] and GlimmerHMM [16], respectively. Furthermore, users can continue with subsequently analysis using (a) all clustered sequences, (b) the Open Reading Frames (ORFs) sequences predicted by gene finders or (c) both.

To estimate G + C content and codon usage, STINGRAY uses the EMBOSS Geecee and Cusp packages [17], respectively. Clusters or ORFs are then submitted to standalone BLAST [19] for similarity searches against user-defined datasets, downloaded and updated by the server administrator, using an intuitive interface.

To use protein section of STINGRAY workflow, users need to upload the amino acid sequences in FASTA format and the system will consider each entry as a unique sequence. It is important to mention that STINGRAY automatically recognizes the project type (nucleotide or protein).

**Figure 2:**
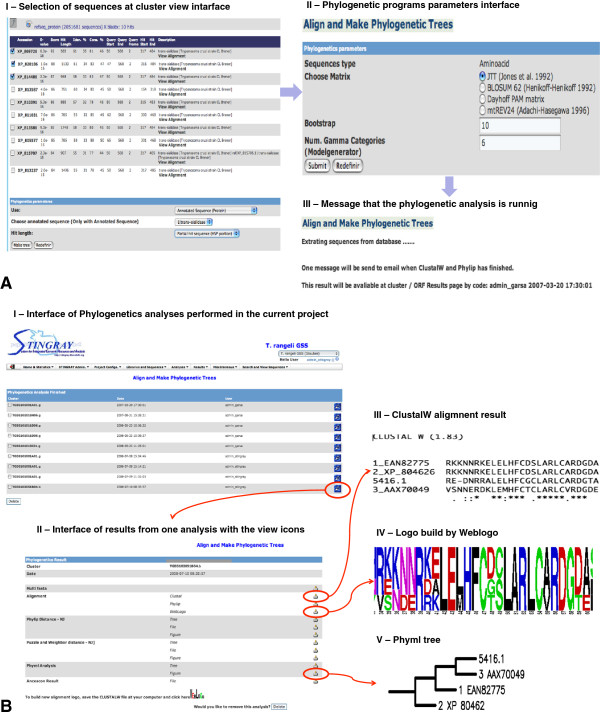
**Phylogenetic workflow of STINGRAY. A.I)** Blast hits and project sequence selection; **A.II)** interface to define the evolutionary and phylogenetic parameters used by Phylip; **A.III)** phylogenetic progress analyses information. When the phylogenetic execution is concluded, the previous code is presented in cluster/ORF view page **(B.I)**; list of all phylogenetic results **(B.II)**; ClustalW alignment **(B.III)**; alignment logo was performed by WebLogo program [24] (B.IV) and phylogenetic tree was performed by Phylip program **(B.V)**.

STINGRAY offers to user a phylogenetic module that all three steps typically necessary for molecular phylogenetic analysis, (1) retrieval/inference of homologous sequences, (2) creation of multiple sequence alignments and, (3) phylogenetic tree construction can be performed in STINGRAY. Besides, the system allows the user to infer phylogenetic trees using either full cluster, ORF or high-scoring segment pairs (HSP) obtained automatically by BLAST, then perform multiple sequence alignments generated using ClustalW [30], MAFFT [22] or ProbCons [23] packages. The resulting alignments can be presented in ClustalW, PHYLIP and/or WebLogo formats. Phylogenetic trees are built using SeqBoot, Dnadist, Protdist, Neighbor and Consense software from PHYLIP package [26], as well as Weighbor [27] or Ancescon [25] algorithms. The generated trees are presented in PHYLIP, NEXUS and NEWICK formats, which are available for users visualization and download (Figure 2).

### STINGRAY schema

STINGRAY uses the MySQL Database Management System (DBMS) to store all data in order to improve the performance, data security and management. A relational schema was specially designed to register and straightforward future reference of all data produced by the workflow execution. The schema is able to register data from projects and their users and also permits to maintain data about project-specific configuration and access restrictions. In addition, the STINGRAY data schema also provides some data provenance. For instance, the Cluster table, in the core of the STINGRAY data schema (Additional file 3), records the cluster sequence under investigation and these are connected to the reads used to assemble it by the provenance registered in the Clusters_Reads table. The clustering/assembly software execution (e.g. CAP3, MIRA or VELVET) is also registered in the Clustering table.

Furthermore, each BLAST analysis is registered in the "Blast_Search" table, which stores information about the parameters values used for that BLAST analysis, such as the sequence database (e.g. NCBI nr, Swissprot), BLAST algorithm (e.g. blastx, blastn) and all BLAST similarity feature, as hit sequences, accessions, score, e-value, consensus sequences, identity and positive values among others are sorted in the Blast_Hit table. So it is possible to link the results with a specific BLAST analysis. The Additional file 4 shows the complete relational schema.

### Functional annotation

Once assembled, sequence clusters are generated and used for subsequent analyses. All sequences and their analyses results can be viewed by a intuitive Web interface (Additional files 5 and 6), such as the Cluster View page where all data analyses for a chosen cluster are summarized (Additional file 7). Users can also compare sequences from a specific library, compare common sequences among all libraries or even obtain library-specific sequences. BLAST [19], InterProScan [31], PSI-BLAST [19], HMMER [31], tRNA-Scan [18], WolfPsort [21], SignalP [20], Gene Prediction and Automatic Function Prediction (AFP) (by using Glimmer [15] or GlimmerHMM [16]) results are presented in specific tables in a unique interface that holds all necessary information for user analysis (Additional file 8). Besides that, in this interface, the user select sequences to perform phylogenetic analysis as described before (Figure 2). Another important feature provided by STINGRAY is to allow ontology-based functional annotation using Gene Ontology (GO) terms ( http://www.geneontology.org/) as part of AFP. This feature was implemented based on similarity results with databases like Seqdblite (GO), UniProtKB (Swiss-Prot and TrEMBL) [32], and InterPro [33] results. Briefly, the GO descriptions, associated to sequences from each quoted databases, are used for semi-automatic annotation of clusters, ORFs or proteins. The methodology that scores the terms through accordance and distance methods was incorporated into STINGRAY [34]. The system suggests which terms are more "related" to the protein sequence being analyzed, allowing the user to define the best functional annotation for a sequence during the manual annotation process.

For manual annotation, the user must inform the region of the cluster that corresponds to a coding sequence (CDS) or select one ORF in the list of the cluster. STINGRAY then estimates the G + C content, predicts the amino acid sequence for CDS, sub-cellular location (using Wolf-Psort) and peptide cleavage signals (using SignalP). When available, information about (i) Enzyme Commission Code (Union of Biochemistry and Molecular Biology), (ii) Monica Riley classification, (iii) most similar species, (iv) BLAST similarity, (v) domain, and (vi) notes about sequences, are automatically included as part of the annotation process (Additional file 8). After one sequence has been annotated by AFP, the user can manually verify the results through an interface to confirm or update the automatic annotation.

Since STINGRAY is a multi-user system, the confidentially and maintenance integrity of the data are important. To achieve this level of security only "project administrator" and "write" users can modify and run the programs for sequence annotation. Nevertheless, only project administrators have permission to remove data.

Once a project in STINGRAY is finished/published, the project administrator allows, upon user request, the data and analyses to become public, and then the scientific community (or "read" users) can view some project details (Additional file 9), statistics (Additional file 10) and graphics.

A common concern in sequencing projects, especially EST and GSS projects, is the GenBank submission of the annotated sequences. In order to facilitate and enable the submission, STINGRAY provides an intuitive interface where sequences and data are formatted according to the GenBank requirements. Since the system does not perform automatic submission, the formatted file must be submitted to GenBank by the project administrator *per se*.

### Pre-assembly and automatic functional prediction

In order to test the STINGRAY workflow, bacterial genomes from *Escherichia coli* K12, *Neisseria meningitides, Streptococcus pneumoniae* GA17457, and a eukaryotic genome: *Phlebotomus papatasi* (NCBI Sequence Read Archive number SRX000353, ERX005963, SRX028097 and SRX027131, respectively) were processed, pre-assembled then AFP was performed. The time of pre-assembly, GO-based annotations, and numbers of contigs obtained by pre-assembly for each of the four data sets are listed in Table 2.

**Table 2:** Pre-assembly and automatic functional prediction test

**Organism**	**Genome size**	**SRA**^**1**^	**Sequencing technology**	**Total reads**	**N° of contigs**	**Time of pre-assembly**^**6**^	**Number of sequences with one GO-based annotations at least**			
							**MF**^**2**^	**BP**^**3**^	**CC**^**4**^	**WA**^**5**^
*E. coli* K12	4,7 Mb	SRR001354	SOLiD	25162805	11011	30 min	1593	1577	1562	9328
*N. meningitidis*	2 Mb	ERR015596	Illumina	5418859	4815	3 h 4 min	1055	1055	995	2812
*S. pneumoniae* GA17457	2 Mb	SRR068304	454 GS FLX	252646	11317	2 h 52 min	5899	5942	5393	5340
*P. papatasi*	~170 Mb	SRR066482	454 Titanium	498629	9836	5 h 18 min	554	553	458	9993

1 - SRA: Sequence Read Archive.

2 - MF: Molecular Function.

3 - BF: Biological Process.

4 - CC: Cellular component.

5 - WA: Without annotation.

6 - Pre-assemblies were performed on Intel 8 Core Server (1860mHz) and 16Gb RAM.

## Conclusions

Nowadays, STINGRAY is hosting more than 20 different projects, among them the *T. vivax* (GSS and EST) [35], *Bothrops jararaca* (EST) [36], *Lutzomyia longipalpis* (EST) [37,38], *Taenia solium* (EST) [39] and *Trypanosoma rangeli* (GSS, EST and ORESTES) [40]. The main advantage of STINGRAY over related systems is its larger and flexible workflow on which advanced users or annotators are able to fine-tune the parameters of some programs to extract the maximum of valuable information and knowledge from their sequences.

The STINGRAY pipeline is able to manipulate both Sanger and NGS sequence data in the same project (Table 3), whereas other (recently developed) pipelines do not longer center on Sanger technology. Since Sanger sequencing is still widely used, a system being able to deal with these two technologies should be seen as an advantage. Assembly quality is strongly dependent on quantity (coverage) and quality of data/reads as well as "fine tuning" of the many parameters available in the genome assembler software, then while the pre-assembly performed was done with the only purpose to illustrate the STINGRAY functionalities, the results obtained showed to be a good starting point for additional and robust assembly process.

**Table 3:** Features comparison between Stingray and other annotation pipelines

**Features**	**STINGRAY**	**RATT**	**Artemis**	**WEP**	**NGSPE**
Preproccesing sequencing output data files	X			X	X
Proccesing Sanger output files	X				
Proccesing NGS output files	X			X	X
Gene prediction	X	X	X		
Similarity search blast	X		X		
Similarity search Hmmer	X		X		
Similarity search RPSBlast	X		X		
Similarity search Interpro	X				
Similarity search PSIBlast	X				
Homologs identification	X	X			
Phylogeny analysis	X				
Codon usage analysis	X				
tRNA prediction	X				
Manual annotation	X		X		
Semi-automatic annotation	X				
Automatic annotation	X	X		X	X
Friendly interface	X		X		
Web platform	X			X	
Use of SGBD	X			X	
Applet			X		
Browse genome visualization			X		
Jalview visualization			X		
Generate GBFF file	X		X		
Generate SeqIn file	X		X		

Furthermore, STINGRAY offers a complete annotation pipeline, allowing the user to perform automatic, semi-automatic and manual annotation, while others pipelines like RATT [41], WEP [6] and NGSPE [7] perform only automatic annotation. STINGRAY also allows the user to edit annotations being, along Artemis [42,43], the unique systems with such feature. Up to the current version, STINGRAY is the only pipeline which allows the use of Intepro Search, Phylogeny analysis, Codon Usage Analysis and tRNA sequence prediction in a integrated way, then being a web-based platform with friendly interface is a plus.

Due the open-source nature, future developments and improvements such as the incorporation and analysis of DNA-, RNA- or Methyl-sequencing data as well as analysis of sequence, functional or structural variants are possible. Also, the use of "cloud-based" applications as part of the STINGRAY workflow, either using private clouds or even commercial ones as Amazon’s ( http://aws.amazon.com/ec2/), are being considered. In the current context of larger high-throughput sequence generation, the use of cloud computing is the way forward. Larger and improved database schemas as GUS (Genomics Unified Schema) ( http://www.gusdb.org/) could be potentially used to content different datasets and sequence features. Data integration using LOD (Linked Open Data) technology is also planned for the next version, as it is now clear that connecting local data with many other sources (curated, non-curated or even complementary) in the LOD cloud ( http://lod-cloud.net/versions/2007-11-10/lod-cloud.png) might help to accelerate knowledge extraction. Online documentation for installation using STINGRAY and technical information are available at http://stingray.biowebdb.org and on Additional File 11.

## Availability and requirements

**Project name:** STINGRAY (BiowebDB)

**Project home page:**http://sourceforge.net/projects/stingray-biowebdb/

**Operating system(s):** Unix

**Programming language:** Perl

**Other requirements:** Perl, Apache, MySQL

**License:** GNU GPLv2.

**Any restrictions to use by non-academics:** license

### Availability of supporting data

The data set(s) supporting the results of this article is (are) included within the article (and its additional file(s)).

## Abbreviations

AFP: Automatic function prediction; BLAST: Basic local alignment search tool; CDS: Coding sequences; CGI: Common gateway interface; DBMS: Database management system; DDBJ: DNA Data Bank of Japan; EMBOSS: The European Molecular Biology Open Software Suite; EST: Expressed sequence tags; GARSA: Genomic analysis resources for sequence annotation; GATO: Gene annotation tool; GSS: Genomic sequence survey; GO: Gene Ontology; GUS: Genomics unified schema; LOD: Linked open data; MPI: Message passing interface; NCBI: National Center for Biotechnology Information; NFS: Network file system; NGS: Next generation sequencing; ORESTES: Open reading frame ESTs; ORF: Open reading frames; PERL: Practical extraction and report language; PSI-BLAST: Reversed position specific BLAST; RPS-BLAST: Position specific iterative BLAST; SABIA: System for annotation bacterial (genome) integrated annotation; SQL: Structured query language; STINGRAY: System for integrated genomic resources and analysis; tRNA: Ribonucleic acid transporter.

## Competing interests

All other authors declare that they have no competing interests.

## Authors’ contributions

GW, DAT and DRL were responsible for programming, development and tests with Sanger data. KACSO participated in the programming and development of some bioinformatics tasks and drafted the manuscript. RJ, ACBR and VEE participated in the assembly development and tested the system. CMP, ANP, ECG, MCC, MLMC, MM, AMRD conceived of the study, and participated in its design and coordination and helped to draft the manuscript. All authors read and approved the final manuscript.

## Supplementary Material

Additional file 1 **Screenshot from configuration interface.** With this intuitive interface the system manager can configure all programs paths and options of software include on STINGRAY workflow, as well as some project parameters. Click here for file

Additional file 2 **Schema of FIOCRUZ servers where the STINGRAY is installed.** To improve STINGRAY performance the system platform (i.e. CGI/Perl scripts) was installed on the web-server and software as Phred, CAP3, BLAST, InterProScan, among others were installed on "process server" and MySQL were located on database server. The requested program stared by user STINGRAY on web server is forward to the process server using in-house scripts and after the program has finished the output file located on Network File System (NFS) partition is parsed and the results are stored at MySQL database. Click here for file

Additional file 3 **The core of relational STINGRAY database schema.** This figure shows the resume of relational STINGRAY database schema. The boxes represent the SQL tables and the lanes the relation between the tables. Click here for file

Additional file 4 **The complete STINGRAY database schema.** This figure shows the complete relational STINGRAY database schema. The boxes represent the SQL tables and the lanes the relation between the tables. Click here for file

Additional file 5 **Screenshot of search sequence interface.** In this interface the users can search sequences by the identification of the reads, clusters or ORF or even by BLAST/InterPro/HMMER, annotations or Gene Ontology descriptions. Click here for file

Additional file 6 **Screenshot of BLAST results search interface.** Using this interface the user can view the all similarity BLAST results. Notice the other results interfaces are available at the upper menu. Click here for file

Additional file 7 **Screenshot of cluster view interface.** This intuitive interface shows all cluster features, like length, reads and similarity results obtain by BLAST, InterProScan and HMMER results. The ORF view interface is similar. Click here for file

Additional file 8 **Screenshot of annotation (CDS) interface.** This interface allowed user to annotate the sequence and insert other important information. Click here for file

Additional file 9 **Screenshot of a current available project.** This is the specific project page, with the information about the project and number of sequences. Click here for file

Additional file 10 **Statistic reports interface screenshot.** In this interface the user can view the summary of the current project data. Click here for file

Additional file 11 **STINGRAY source code.** All scripts and web pages needed to setup STINGRAY are available in this compressed file. Click here for file
