# Supplementary figures and images for: STINGRAY: system for integrated genomic resources and analysis
Source: BMC Res Notes. 2014 Mar 7;7:132. doi: 10.1186/1756-0500-7-132 (PMC4015962; doi:10.1186/1756-0500-7-132)

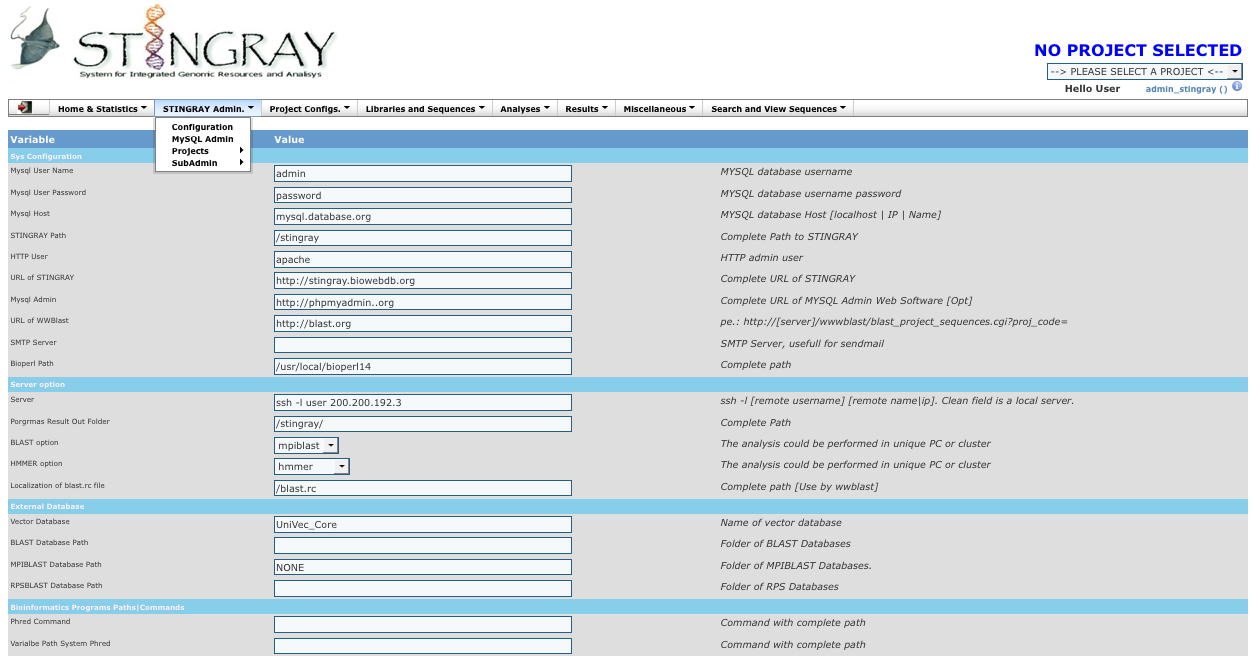

Supplement: Additional file 1 — Screenshot from configuration interface. With this intuitive interface the system manager can configure all programs paths and options of software include on STINGRAY workflow, as well as some project parameters. [file 1756-0500-7-132-S1.png]

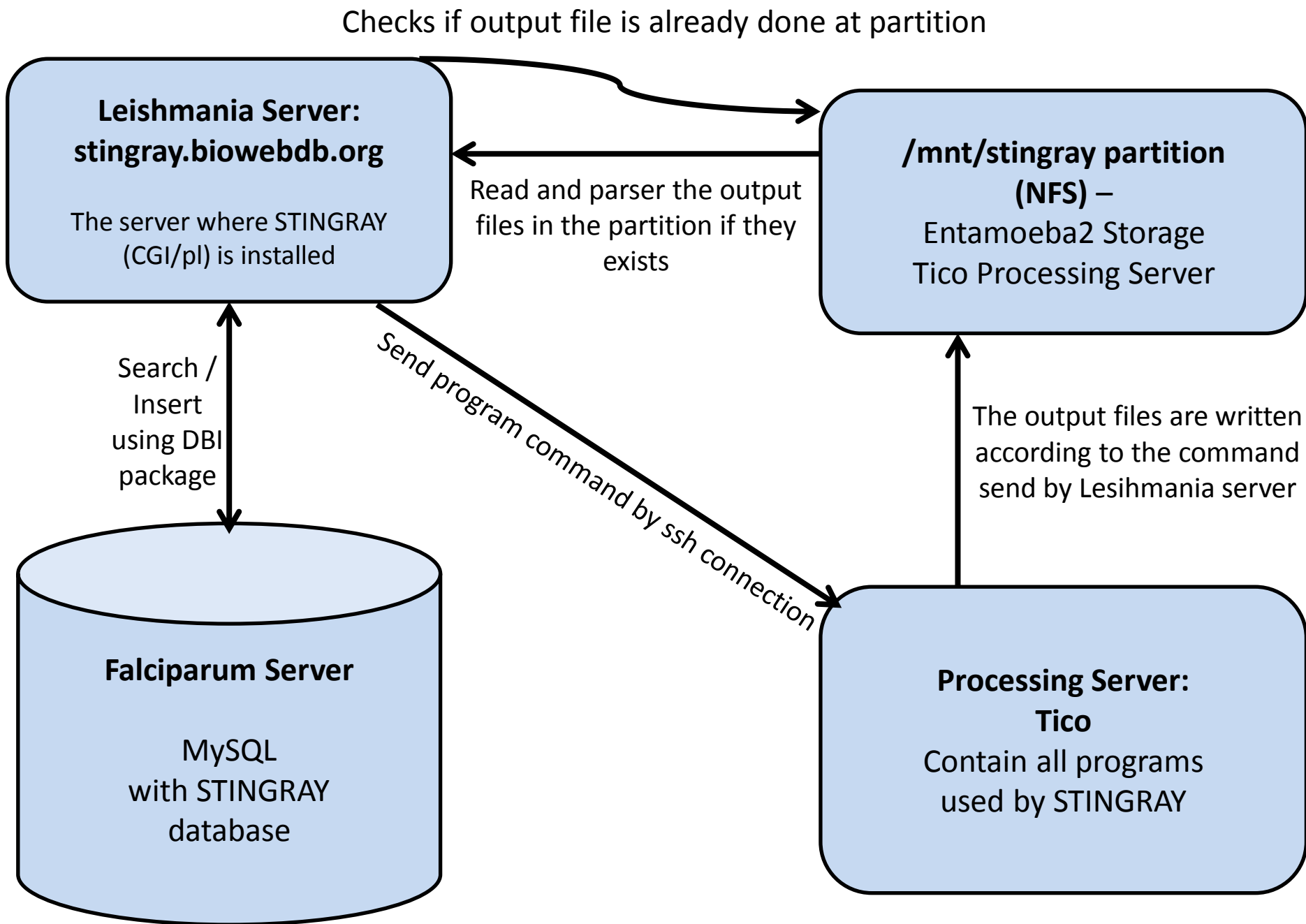

Supplement: Additional file 2 — Schema of FIOCRUZ servers where the STINGRAY is installed. To improve STINGRAY performance the system platform (i.e. CGI/Perl scripts) was installed on the web-server and software as Phred, CAP3, BLAST, InterProScan, among others were installed on "process server" and MySQL were located on database server. The requested program stared by user STINGRAY on web server is forward to the process server using in-house scripts and after the program has finished the output file located on Network File System (NFS) partition is parsed and the results are stored at MySQL database. [file 1756-0500-7-132-S2.pdf]

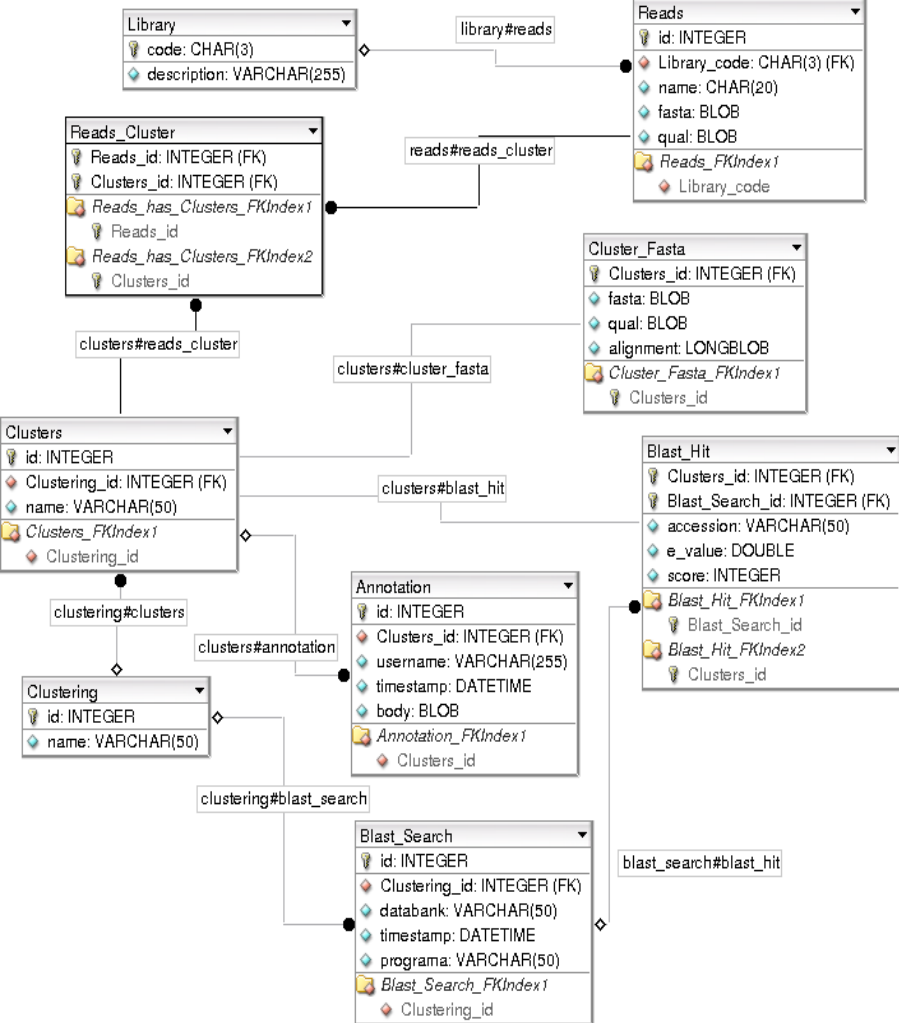

Supplement: Additional file 3 — The core of relational STINGRAY database schema. This figure shows the resume of relational STINGRAY database schema. The boxes represent the SQL tables and the lanes the relation between the tables. [file 1756-0500-7-132-S3.pdf]

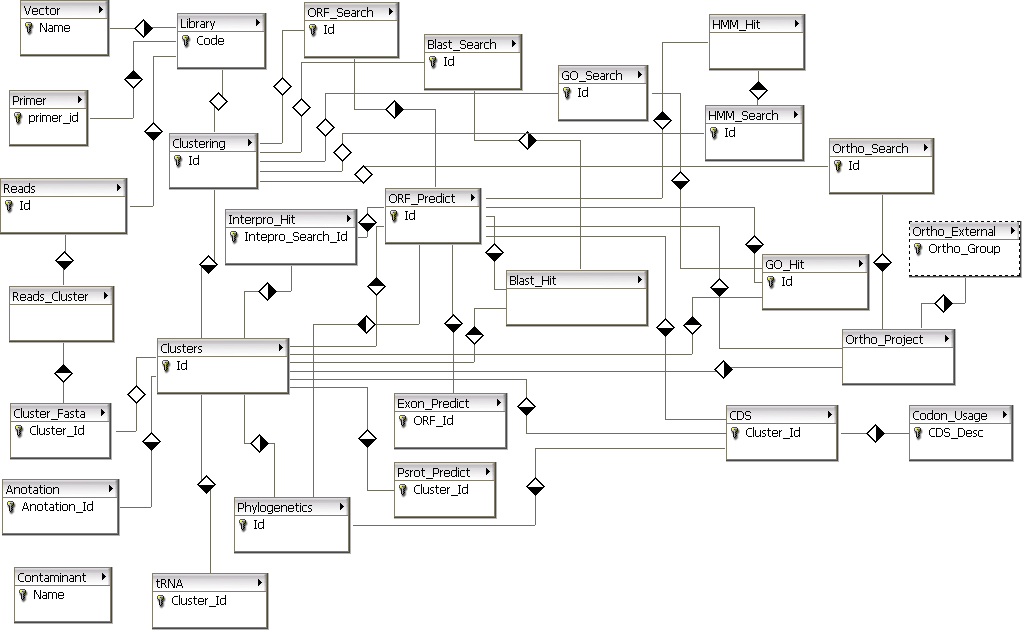

Supplement: Additional file 4 — The complete STINGRAY database schema. This figure shows the complete relational STINGRAY database schema. The boxes represent the SQL tables and the lanes the relation between the tables. [file 1756-0500-7-132-S4.png]

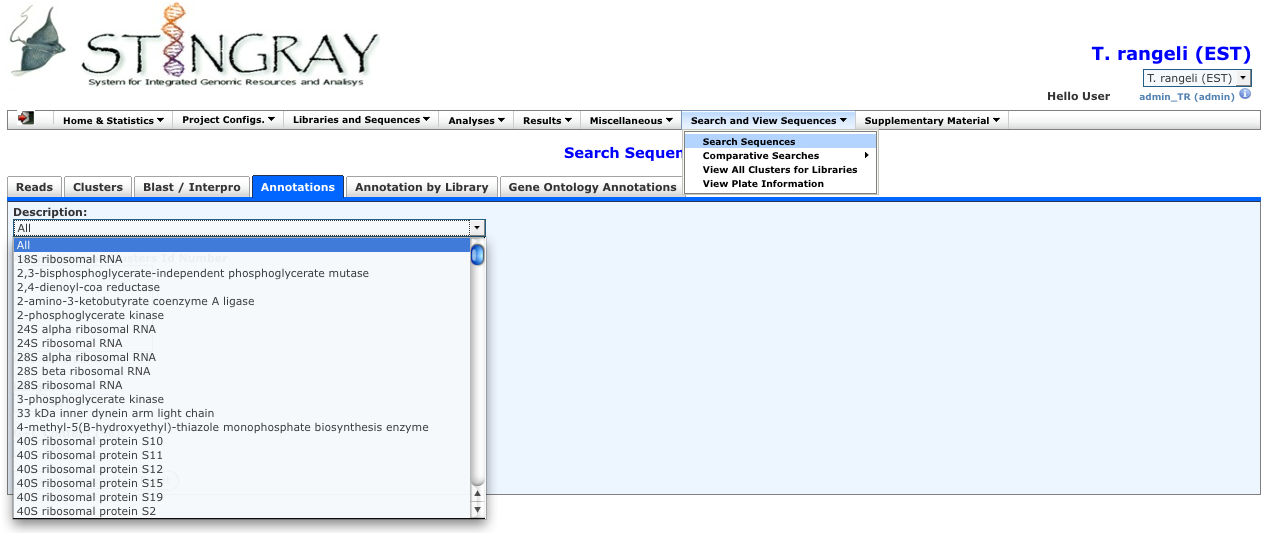

Supplement: Additional file 5 — Screenshot of search sequence interface. In this interface the users can search sequences by the identification of the reads, clusters or ORF or even by BLAST/InterPro/HMMER, annotations or Gene Ontology descriptions. [file 1756-0500-7-132-S5.png]

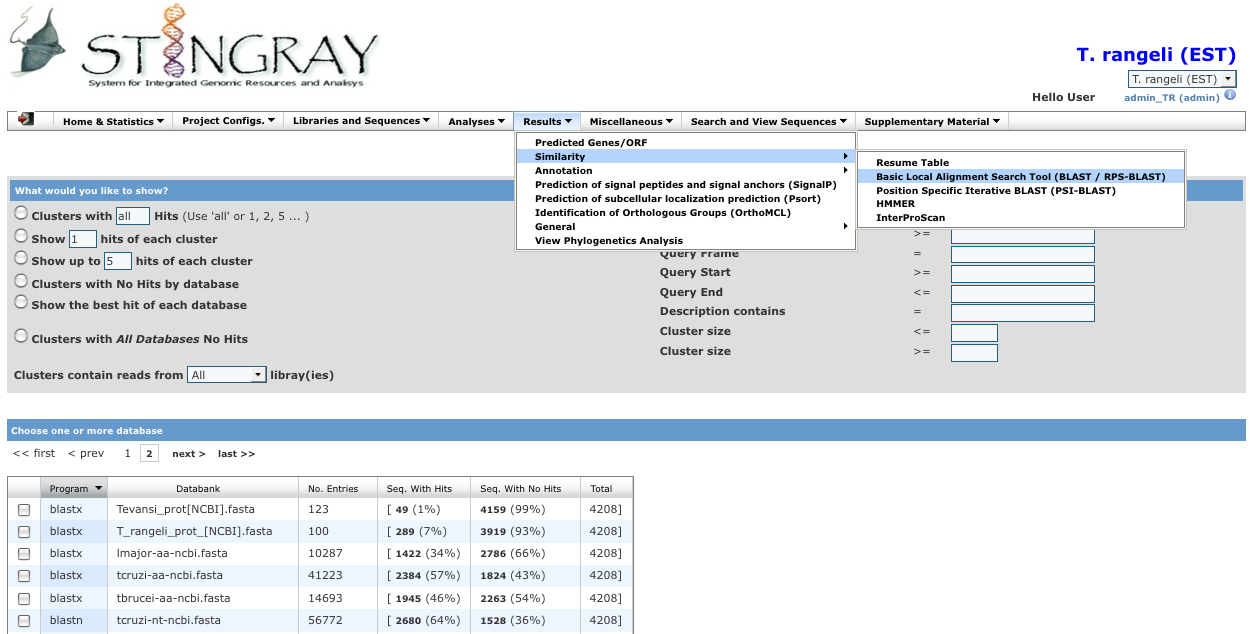

Supplement: Additional file 6 — Screenshot of BLAST results search interface. Using this interface the user can view the all similarity BLAST results. Notice the other results interfaces are available at the upper menu. [file 1756-0500-7-132-S6.png]

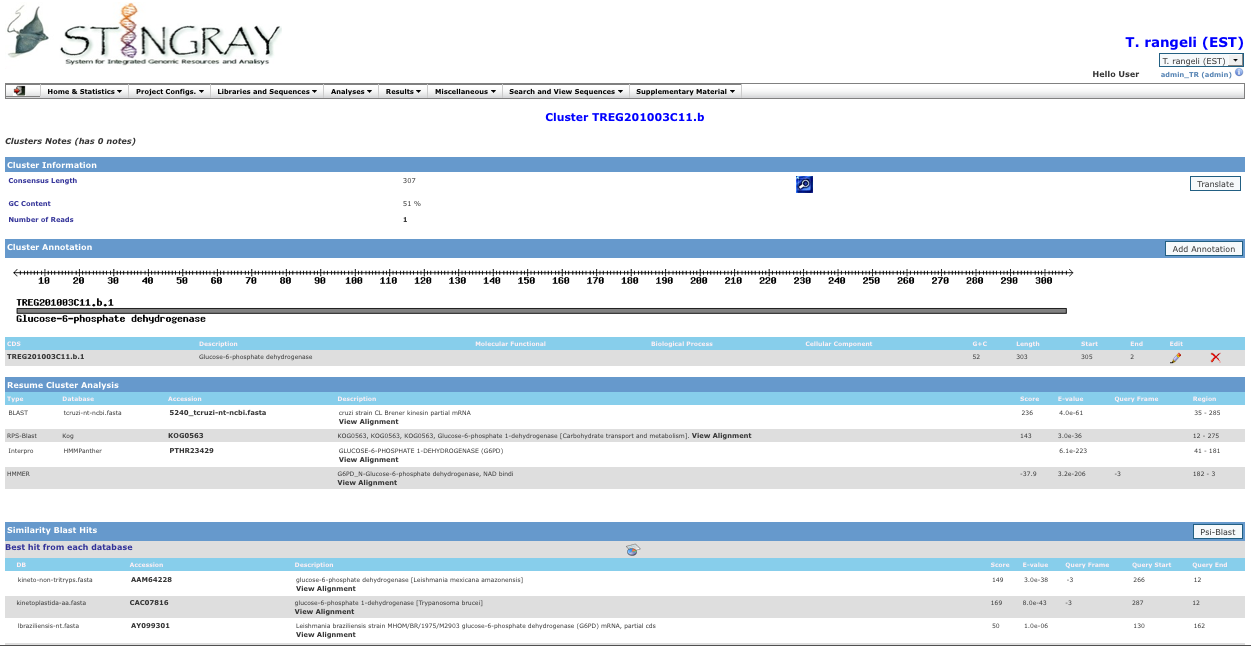

Supplement: Additional file 7 — Screenshot of cluster view interface. This intuitive interface shows all cluster features, like length, reads and similarity results obtain by BLAST, InterProScan and HMMER results. The ORF view interface is similar. [file 1756-0500-7-132-S7.png]

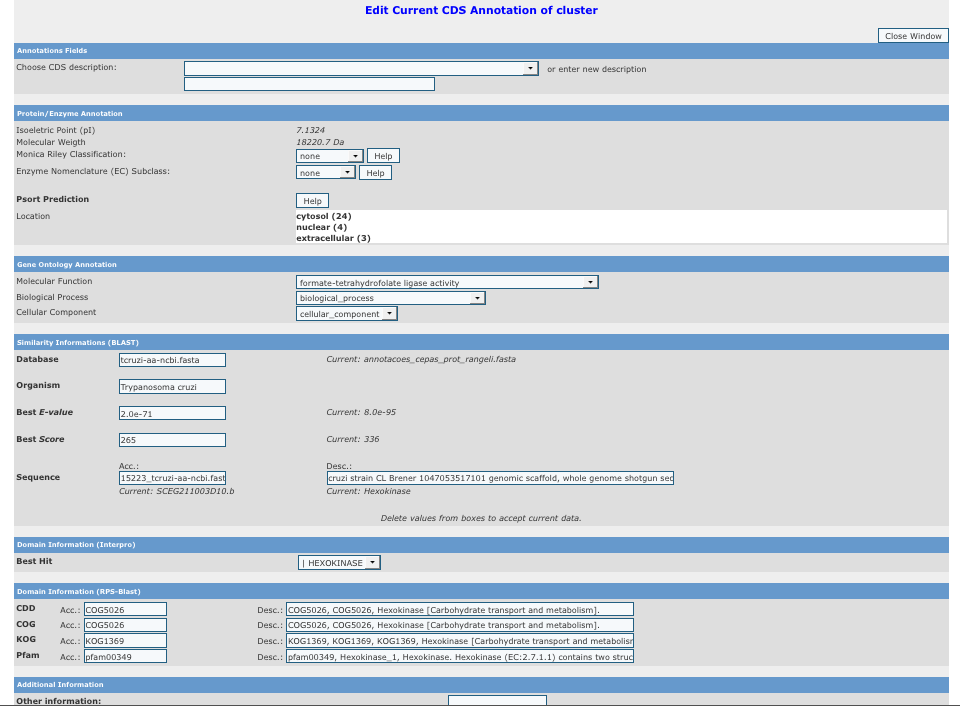

Supplement: Additional file 8 — Screenshot of annotation (CDS) interface. This interface allowed user to annotate the sequence and insert other important information. [file 1756-0500-7-132-S8.png]

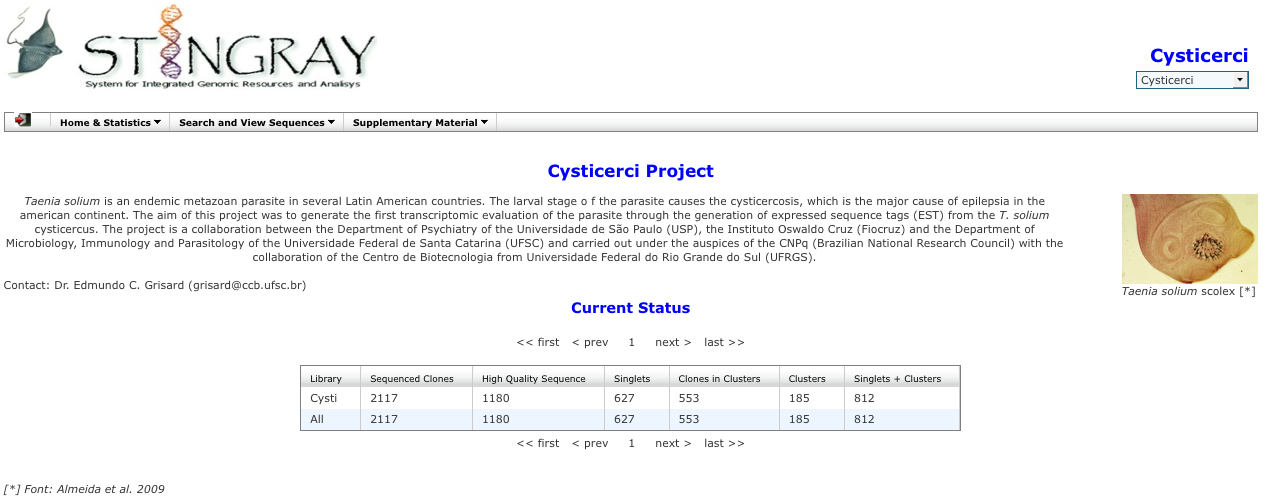

Supplement: Additional file 9 — Screenshot of a current available project. This is the specific project page, with the information about the project and number of sequences. [file 1756-0500-7-132-S9.png]

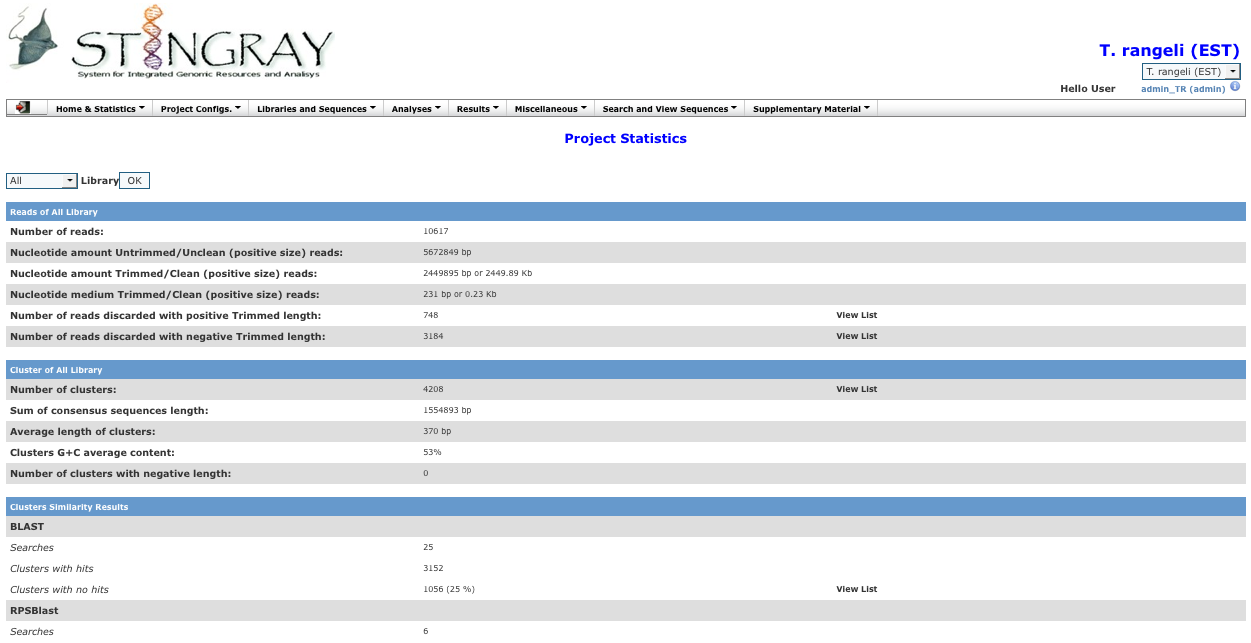

Supplement: Additional file 10 — Statistic reports interface screenshot. In this interface the user can view the summary of the current project data. [file 1756-0500-7-132-S10.png]
